# Thermal Decomposition of Kraft Lignin under Gas Atmospheres of Argon, Hydrogen, and Carbon Dioxide

**DOI:** 10.3390/polym10070729

**Published:** 2018-07-03

**Authors:** Qiangu Yan, Jinghao Li, Jilei Zhang, Zhiyong Cai

**Affiliations:** 1 Department of Sustainable Bioproducts, Mississippi State University, Mississippi State, MS 39762, USA; yanqiangu@gmail.com; 2 U.S. Department of Agriculture, Forest Service, Forest Products Laboratory, Madison, WI 53726, USA; jli@fs.fed.us

**Keywords:** kraft lignin, thermal decomposition, H_2_ atmosphere, CO_2_ atmosphere

## Abstract

The behaviors of thermal decomposition of kraft lignin under three different gases (Ar, CO_2_, or H_2_) were analyzed and compared using a temperature-programmed decomposition-mass spectrometry (TPD-MS) system. Experimental results indicated that Ar atmosphere produced the highest yield of solid chars, while H_2_ atmosphere generated the highest yield of liquids and CO_2_ atmosphere had the highest yield of gases. TPD-MS results showed that H_2_ atmosphere was consumed at the temperature range from 205 to 810 °C and CO_2_ atmosphere was consumed at the temperature range from 185 to 1000 °C. The H_2_ promoted the cleavage of lignin side chains and significantly enhanced the formation of CH_4_, C_6_H_6_, HCHO, C_6_H_5_OH, CH_3_OH, and tars. The percentages of water in produced liquids were 90.1%, 85.3%, and 95.5% for Ar, H_2_, and CO_2_ as atmosphere, respectively. The H_2_ yielded more organic chemicals in produced liquids compared to the other two gases. The observed organic chemicals were mainly acetic acid, phenols, ketones, alcohols, aldehydes, and esters. BET surface areas of solid products were 11.3, 98.5, and 183.9 m^2^/g for Ar., H_2_, and CO_2_ as the atmosphere, respectively. C–H–O–N–S elemental and morphology analyses on solid products indicated that the lowest carbon content and the highest oxygen content were obtained if Ar atmosphere was used, while H_2_ and CO_2_ yielded more carbon in final solid products. Solid products obtained under CO_2_ or H_2_ atmosphere contained sphere-shaped nanoparticles.

## 1. Introduction

Lignin is the second major component of lignocellulosic biomass and the most abundant aromatics biopolymer resource on earth [1,2]. Lignin is a byproduct produced from wood delignification and the pulping process; the global annual production of lignin is approximately more than 70 million metric tons [3,4]. The kraft process is the most widely used pulping process and kraft lignin accounts for more than 90% of the world’s chemical pulp lignin production [5]. However, only 1–2% of lignin is isolated from wood pulping for commercial applications; the majority is burned onsite for heat and pulping chemical recovery [6]. With the increasing awareness of environmental issues and the depletion of fossil fuels, there is a tremendous research interest in utilizing biomass like lignin for the production of sustainable/renewable fuels and chemicals [7,8,9,10,11,12] and carbon-based nanomaterials like active carbons [13,14], carbon fibers [15], templated carbon [16], and graphene [17] through thermal conversion technologies.

The thermal decomposition of lignin has been extensively studied under an inert atmosphere using various techniques, such as thermogravimetric analysis (TGA) [18,19], Fourier-transform infrared spectroscopy (FTIR) [19,20], differential scanning calorimetry (DSC) [21], gas chromatography (GC)-mass spectrometer (MS) [22], and GC-nuclear magnetic resonance (NMR) [23], or the coupling of these characterization techniques [24,25]. The thermal decomposition process results in final products like volatiles, including low molecular gases (CO_2_, H_2_O, CO, H_2_) [18], light hydrocarbons (CH_4_, C_2_H_6_), aromatics, and oxygenates (CH_3_OH, HCHO, and phenols), as well as liquids and solid char. The key factors affecting the property and distribution of the final products from thermal decomposition of lignin are heat treatment temperature, types of feedstock, feedstock particle size, and heating rate [26,27,28]. In addition, atmosphere used in the heating process affects the distribution of final products [29]. Up to date, most of literature reports the findings of thermal decomposition of lignin performed under inert atmospheres like He, Ar, or N_2_. Limited reports were found in relation to the thermal decomposition of lignin under reactive atmospheres such as CO_2_ or H_2_ [30].

Gasification of biomass under CO_2_ atmosphere can convert solid carbon to CO-rich gas for the production of fuels [31]. In addition, hydrogasification of coal under H_2_ atmosphere can significantly increase the yields of tar and liquid fuels [32]. Further studies on using different atmospheres such as CO_2_ or H_2_ for the decomposition of lignin can help provide better understanding of the mechanism involved in various thermal conversions of lignin. Therefore, the main purpose of this study was to investigate the effects of various gas atmospheres on the thermal decomposition process of kraft lignin. Specifically, the effect of three different gases (Ar, CO_2_, and H_2_) on the yields of liquid, noncondensable gas, and solid carbon products, the formation of all volatiles and liquids, and the structures of solid carbon products of a thermal decomposition process of kraft lignin were investigated.

## 2. Materials and Methods

### 2.1. Materials

Kraft lignin (BioChoice, Plymouth, NC, USA) was supplied by Domtar Corp (Fort Mill, SC, USA). The proximate moisture analysis was carried out following ASTM D4442-07 standard [33]. According to the specification from Domtar, the BioChoice product contained 97.1 wt % of lignin. The ash content of lignin (1.65 wt %) was measured according to ASTM D1102 [34]. Lignin elemental analysis was carried out on a PE 2400 CHNS Elemental Analyzer (PerkinElmer, Waltham, MA, USA). The oxygen content was evaluated by the difference.

### 2.2. FTIR Spectroscopy

The FTIR spectrum of lignin was recorded with the PerkinElmer attenuated total reflection (ATR) spectrometer (PerkinElmer, Waltham, MA, USA) at a resolution of 2 cm^−1^ for 10 scans in 450 to 4000 cm^−1^ range to determine structural changes of kraft lignin during thermal decomposition.

### 2.3. Thermal Decomposition

The thermal decomposition of kraft lignin was performed in a fixed-bed reactor system composed of a stainless tubular reactor (1-inch O.D.) and condenser. For each experimental run, 5 grams of kraft lignin were loaded into the middle of the stainless tubular reactor. Each of three purging gases, Ar, H_2_, or CO_2_ (purity ≥ 99.99%), was seperately introduced into the system with a flow rate of 80 mL/min for 15 min, followed by raising the temperature to 1000 °C at a ramping rate of 10 °C/min. After being held at 1000 °C for 1 h, the furnace was turned off and allowed to cool to ambient temperature under the purging gas atmosphere. The solid and liquid products were collected from the system and weighed to calculate their mass distributions. The yield of gas products was calculated based on the differences among the three products yielded from the decomposing process.

The gaseous and volatile products released during the decomposition of kraft lignin under different atmosphere were continuously monitored and analyzed through an on-line residue gas ananlyzer (RGA). The signals from the mass spectra of 2, 15, 28, 30, 31, 34, 44, 78, and 94 (*m*/*z*) were identified as the major contributors for specifically evolved gases and volatiles of H_2_, CH_4_, CO, HCHO, CH_3_OH, H_2_S, CO_2_, benzene, and phenol, respectively. Liquid products collected from the condensor were also analyzed and water content in the liquid products was measured using the Karl Fischer Titration. Water in liquid products was removed using isotropic distillation with toluene. The chemical composition of dewatered liquid products was performed using water-free basis using a gas chromatography/mass spectrometry analyzer (Agilent 6890, Agilent, Santa Clara, CA, USA). Elemental and surface area analyses were performed on solid products of kraft lignin thermally decomposed using a CHNS/O analyzer (Perkin Elmer PE2400 series II, PerkinElmer, Billerica, MA, USA) and an automatic adsorption unit (Autosorb–1, Quantachrome, Boynton Beach, FL, USA), respectively. The morphology of solid products was investigated using a scanning electron microscope (JEOL JSM-6500F Field Emission Scanning Electron Microscope, Peabody, St. Louis, MO, USA ) operated with accelerating voltage of 5 kV. All SEM samples were precoated with 10 nm Pt before being introduced into the vacuum chamber.

## 3. Results and Discussion

### 3.1. FT-IR

**Figure 1:**
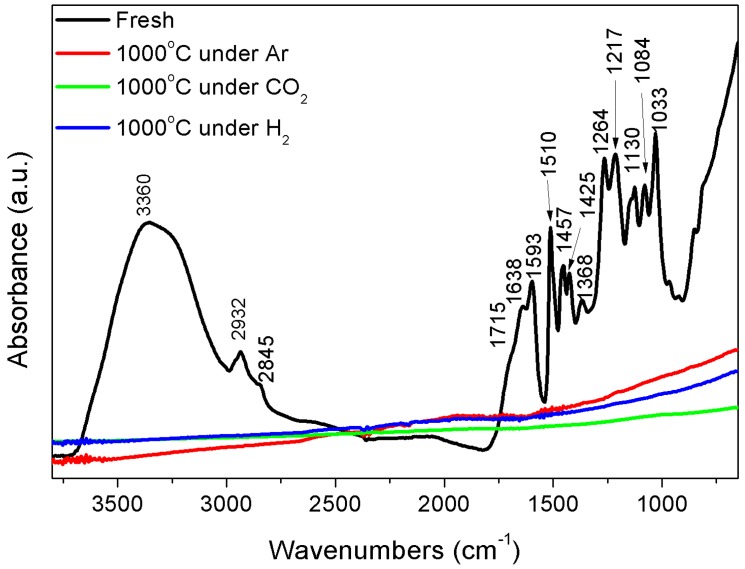
FTIR spectrum of raw and thermally decomposed kraft lignin.

Figure 1 shows the FT-IR spectrum of raw and thermally decomposed kraft lignin. There is a presence of a wide band at 3360 cm^−1^ observed in raw kraft lignin, indicating the presence of hydroxyl groups in phenolic and aliphatic structures (OH stretching vibration). There is also a range of signals around 2932 (C–H stretchings in aromatic methoxyl groups as well as in methyl and methylene groups of side chains) and 2845 cm^−1^ (symmetric C–H stretching in –CH_2_– and tertiary C–H groups) typically found in lignin. The spectral region below 2000 cm^−1^ for raw kraft lignin is more difficult to analyze because most bands with contributions from various vibration modes are complex. In the carbonyl region, weak to medium bands are found at 1715 cm^−1^ that can be associated to unconjugated C=O, at 1638 cm^−1^ related to conjugated carbonyl/carboxyl spectra, and 1595 cm^−1^ corresponds to vibrations in the aromatic ring of lignin plus C=O stretching. A characteristic band at approximately 1510 cm^−1^ corresponds to benzene ring stretching vibrations for softwood lignin (Guaiacyl-G) [35]. The band at 1457 cm^−1^ is assigned to the asymmetric deformation of C–H bonds, while the band at 1420 cm^−1^ corresponds to the vibration of aromatic rings of lignin. The band at 1368 cm^−1^ represents the contribution of OH bendings of hydroxyl groups. A prominent band at 1264 cm^−1^ is assigned to C–O of guaiacyl rings, while the C–O stretching vibration in syringol rings is designated to the peak at 1217 cm^−1^. A band at 1130 cm^−1^ is related to aromatic C–H in-plane deformation in guaiacyl rings, and a 1084 cm^−1^ band is assigned to C–O deformations of secondary alcohols and aliphatic ethers. The spectral region at 1033 cm^−1^ is because of aromatic C–H deformation and C–H out-of-plane vibrations. The FTIR spectra of thermally decomposed kraft lignin demonstrates that almost all the functional groups existing in raw kraft lignin have disappeared after going through the thermal decomposition at a high temperature.

### 3.2. Product Distribution

**Figure 2:**
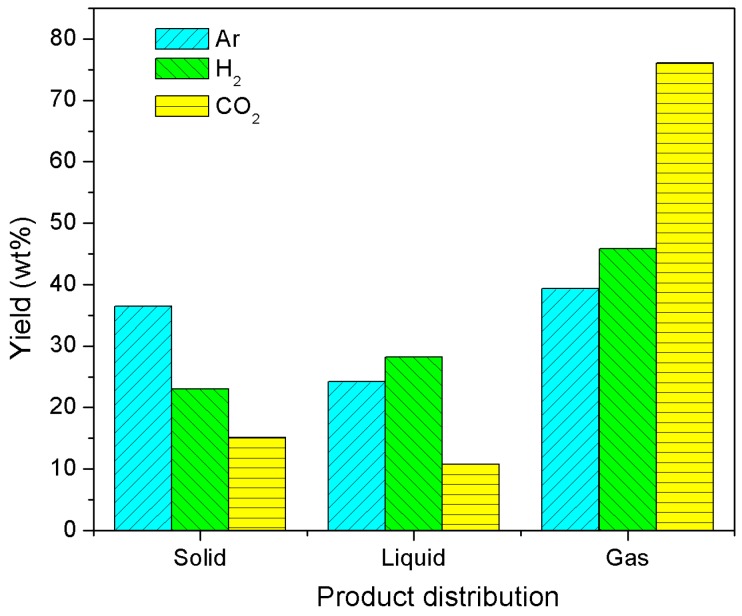
Products (solid carbon, liquid, and noncondensable gas) distribution of kraft lignin thermally decomposed using three different atmospheres at 1000 °C for 1 h.

The summary of product yields (Figure 2) for the thermal decomposition process of kraft lignin indicates that argon atmosphere had the highest solid char yield of 36.5% among the three atmospheres evaluated, followed by H_2_ with a yield of 23.0% and CO_2_ with 15.1%. This can be explained by the fact that more components in kraft lignin were converted to liquid products or gases with reactive atmospheres like H_2_. Thermal decomposition of kraft lignin under H_2_ atmosphere produced 28.4% liquid products, the highest yield among the three atmospheres, followed by 24.2% for Ar and 10.8% for CO_2_ atmospheres. This is probably related to the conversion of oxygen in kraft lignin to water and organic compounds by hydrogen.

Figure 2 demonstrates that the thermal decomposition of kraft lignin under CO_2_ atmosphere yielded the highest weight percentage value of 76.1% for gaseous phase, followed by 39.3% for Ar and 45.8% for H_2_ atmospheres. The thermal decomposition of kraft lignin under CO_2_ atmosphere produced less solid and liquid products and more gaseous products than under Ar and H_2_ atmospheres because kraft lignin and its char are gasified by CO_2_ at high temperature (>600 °C) [31].

### 3.3. Gas Evolution

**Figure 3:**
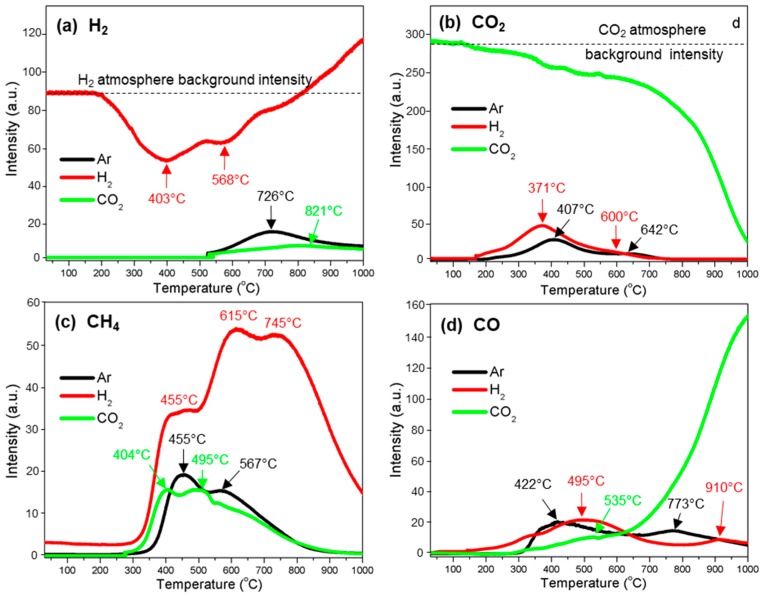
Gas evolution profiles of the thermal decomposition of kraft lignin under argon (Ar), hydrogen (H_2_), and carbon dioxide (CO_2_) atmospheres: (**a**) H_2_; (**b**) CO_2_; (**c**) CH_4_; and (**d**) CO.

**Figure 4:**
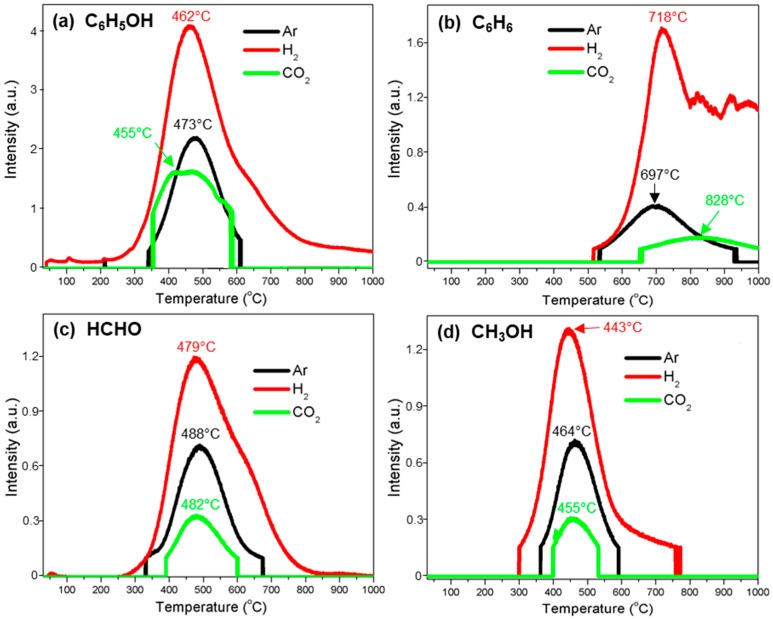
Gas evolution profiles of the thermal decomposition of kraft lignin under argon (Ar), hydrogen (H_2_), and carbon dioxide (CO_2_) atmospheres: (**a**) C_6_H_5_OH; (**b**) C_6_H_6_; (**c**) HCHO; and (**d**) CH_3_OH.

**Figure 5:**
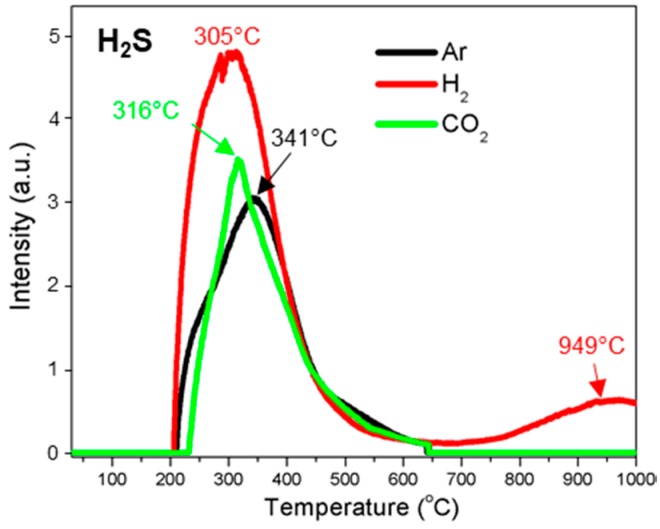
H_2_S evolution profiles of the thermal decomposition of kraft lignin under argon (Ar), hydrogen (H_2_), and carbon dioxide (CO_2_) atmospheres.

Figure 3 summarizes typical gas profiles of H_2_, CO_2_, CH_4_, and CO evolved from the thermal decomposition process of kraft lignin under three different atmospheres of Ar, H_2_, and CO_2_. Figure 4 is the summary of the evolution of major aromatic volatile compounds such as benzene (C_6_H_6_) and phenol (C_6_H_5_OH) from the decomposition of lignin oxygen-containing compounds, and methanol (CH_3_OH) and formaldehyde (HCHO) from the cleavage of lignin side chains. Figure 5 shows the release of hydrogen sulfide (H_2_S) from the thermal decomposition of kraft lignin. Table 1 summarizes the evolution temperature ranges and peak temperatures of all gas phases.

#### 3.3.1. H_2_

Hydrogen presented in kraft lignin is in chemisorbed water as surface functionalities (e.g., carboxyl, phenolic groups) and bonds directly connected to carbon atoms as the part of aromatic or aliphatic structures. The carbon–hydrogen bond (C–H) is very stable but can be broken when heated at a high temperature. The thermal decomposition of kraft lignin in Ar atmosphere eliminates the part of hydrogen via thermal dissociation of C–H in aliphatic CHx (x = 1~3) and aromatic rings [36]. The hydrogen formation temperature under Ar atmosphere (Figure 3a) started at 522 °C and reached its maximum at 726 °C. The H_2_ evolution profile of thermal decomposition of kraft lignin under CO_2_ atmosphere was similar to that of argon atmosphere. However, H_2_ formation was suppressed in the presence of CO_2_ because of the reverse water-gas shift reaction (RWGSR) (CO_2_ + H_2_ = CO + H_2_O [37]). In the reaction, the part of hydrogen from the thermal decomposition reaction of kraft lignin was consumed through RWGSR. The H_2_ evolution curve under the H_2_ atmosphere is dramatically different from these under Ar or CO_2_. H_2_ was firstly consumed by kraft lignin in the temperature ranges of 205–700 °C and then released from kraft lignin at the temperature range from 700 to 1000 °C.

Thermal decomposition of lignin generated various radicals such as phenoxyl (ArO·), methyl (·CH_3_), methoxy (·OCH_3_), hydrogen atom (·H), and other fractional radicals (R) through reaction (1) [38]. Under H_2_ atmosphere, these radicals can react with hydrogen molecules to form stable products like phenols (reaction (2)), methane (reaction (3)), methanol (reaction (4)), and other small compounds through radical processes. The hydrogen consumption was mainly ascribed to reactions (2) to (4) along with the formation of CH_4_, CH_3_OH, HCHO, and C_6_H_5_OH, etc. At temperatures above 700 °C, H_2_ released from the decomposition process was because of the thermal cracking of the C–H bond in lignin chars and organic volatiles.

Lignin → H· + ArO· + ·CH_3_ + ·OCH_3_ + ·CH_2_OH + ·R (1)

ArO· + H_2_ → ArOH (2)

·CH_3_ + H_2_ → CH_4_ (3)

·OCH_3_ + H_2_ → CH_3_OH (4)

#### 3.3.2. CO_2_

The release of CO_2_ exhibited two peaks (Figure 3b and Table 1). CO_2_ mainly originated from the thermal decomposition of carboxyl (–COO^−^) and ester (–CO–O–R) groups [39]. The CO_2_ release peak at lower temperatures (185–583 °C, centered at 407 °C) was because of decarboxylation reactions, while the cleavage of ether groups was predominantly responsible for the CO_2_ evolution at higher temperatures (583–791 °C, centered at 642 °C). The evolution of CO_2_ was significantly promoted under the hydrogen atmosphere compared to that under argon (i.e., two peaks shifted to lower temperatures, i.e., first peak to 371 °C and second peak to 600 °C). These phenomena were caused by the presence of hydrogen, which enhanced the cleavage of lignin side chains [19].

The purging gas CO_2_ was consumed during the thermal decomposition under a carbon dioxide atmosphere (Figure 3b). At low temperatures, CO_2_ might be consumed by active volatiles and solid residues to form various acids and ketones as previously reported [40], while the consumption of CO_2_ at high temperatures (585 to 1000 °C) was because of the gasification reaction along with CO releasing (Figure 4d) as indicated by reaction (5):

C + CO_2_ → 2CO (5)

#### 3.3.3. CH_4_

The evolution of CH_4_ (Figure 3c) has two peaks as previously observed [41,42] (i.e., the first peak has a higher absorbance intensity at around 455 °C while the second peak has a relatively lower absorbance intensity at around 567 °C). The first CH_4_ evolution peak was mainly caused by the cracking of weakly bonded methoxy groups (O–CH_3_) [11,41,42] and the fragmentation of side aliphatic chains [41]. The evolution of CH_4_ at high temperatures was because of the secondary pyrolysis of aromatic volatile intermediates [43,44] and the rearrangement of the lignin carbon skeleton along with CO release to remove the residual oxygen [41,42].

The CH_4_ evolution profile under oxidizing CO_2_ atmosphere was similar to that under argon atmosphere as previously observed [30], while the evolution peaks shifted to lower temperatures of 404 and 495 °C, respectively. This indicated that the presence of carbon dioxide accelerated the cracking of lignin methoxy and aliphatic groups. In addition, the CH_4_ evolution at 800–1000 °C under CO_2_ atmosphere was higher than that under Ar atmosphere, suggesting that the reorganization of the lignin carbon skeleton was promoted by CO_2_.

Three CH_4_ evolution peaks at 455, 615, and 745 °C were observed during the thermal decomposition of kraft lignin under H_2_ atmosphere (Figure 3c). The origin of the first two CH_4_ evolution peaks was similar to those under Ar and CO_2_ atmospheres. The third methane evolution peak occurred at higher temperatures between 680 and 1000 °C. Obviously, this new peak was related to the gasification of solid carbon by hydrogen. During this process, the residual carbon in lignin char reacted with hydrogen to produce methane as indicated by reaction (6):

C(s) + H_2_ ⇌ CH_4_ (6)

#### 3.3.4. CO

The evolution of CO occurred at a wide temperature range from 246 to 1000 °C (Figure 3d); this is because a number of functional groups could contribute to its formation. Two CO evolution peaks were observed under Ar atmosphere at 418 and 770 °C. The lower temperature CO evolution peak with a higher absorbance intensity was because of the rupture of weakly bonded ether (α-*O*-4 and β-*O*-4) bridges and the decarbonylation of the carbon on the C_3_ side-chain [41,42,45], while the higher temperature CO evolution was mainly because of the cleavage of aromatic bonded oxygens (i.e., methoxy and phenolic groups) and the secondary cracking of oxygenate volatiles and tars [20,21,42].

Three CO evolution peaks (Figure 3d) were observed under hydrogen atmosphere, that is, the first was a shoulder peak from 155 to 362 °C, the second was stronger in intensity than the first, ranging from 362 to 780 °C with a maximum temperature at 495 °C, and the third, located at 910 °C, was because of the secondary reactions between volatiles and rearrangement of the char skeleton. Compared to CO formation peaks under argon, the first two peaks under hydrogen shifted to lower temperatures (i.e., the first peak to 362 °C and second peak to 495 °C). This indicated that the evolution of CO was significantly enhanced under a hydrogen flow.

Kraft lignin decomposition under CO_2_ also offered three CO evolution peaks (Figure 3d). The first two peaks were similar to those of argon atmosphere. The third CO peak had a very strong evolution trend at about 1000 °C. This indicates that most of the lignin char residue was consumed by the gasification reactions (Cs + CO_2_ ⇌ 2CO) to produce CO under CO_2_ atmosphere.

#### 3.3.5. Phenol (C_6_H_5_OH)

Under argon atmosphere, the phenol evolution from the thermal decomposition of kraft lignin was observed between the temperatures of 338 and 610 °C with a maximum temperature at 473 °C (Figure 4a). The formation of phenols is related to the dehydration of –OH groups in the alkyl side chain and the cleavage of ether bonds [41]. The C_6_H_5_OH evolution profile under oxidized CO_2_ atmosphere was similar to that under Ar atmosphere, while the evolution peak shifted to a lower temperature at 455 °C because of the promoting effects of CO_2_ on the lignin decomposition process. The C_6_H_5_OH evolution under hydrogen atmosphere occurred at a wide temperature range (239–854 °C) with a stronger intensity peak at 462 °C (Figure 4a). Under hydrogen atmosphere, phenoxyl radicals ArO· can react with a hydrogen molecular to form a phenol molecular and a hydrogen atom, while generated hydrogen atoms can attack a phenoxyl structure in a lignin molecule to form phenoxyl radicals ArO, therefore, more phenol products could be yielded under the hydrogen atmosphere.

#### 3.3.6. Benzene (C_6_H_6_)

Under argon atmosphere, the evolution of benzene was detected in the temperature range from 528 to 938 °C with a maximum temperature at 697 °C (Figure 4b). The formation of benzene was because of the substantial cleavage of hydroxyl groups attached to aromatic rings. The formation of benzene under CO_2_ atmosphere was suppressed at a lower temperature range from 528 to 840 °C, while promoted at a higher temperature range from 840 to 1000 °C. Lignin aromatic compounds tended to be oxidized by CO_2_ [11], and as a result, the formation of benzene was limited. On the other hand, the gasification of lignin char residues was responsible for the high evolution intensity of benzene in the temperature range from 840 to 1000 °C.

The evolution of benzene under a hydrogen flow was detected in the temperature range from 510 to 1000 °C with a maximum temperature at 718 °C. Moreover, the evolution of benzene reached a high and steady level when the temperature was above 799 °C because the formation of polycyclic aromatic hydrocarbons (PAHs) at high temperatures was promoted by hydrogen, while more C_6_H_6_ was generated simultaneously from the secondary cracking of PAHs.

#### 3.3.7. Formaldehyde (HCHO)

The formation of HCHO under argon atmosphere mainly occurred between 329 and 678 °C with a maximum temperature at 488 °C (Figure 4c). The fragmentation of lignin side chains, for example, the cleavage of C_β_–C_γ_, is responsible for the HCHO evolution [39,40]. At carbon dioxide atmosphere, the HCHO evolution was suppressed because lignin side chains tended to be oxidized by CO_2_ to form acids rather than HCHO.

The evolution of HCHO under hydrogen appeared at a very wide temperature range from 249 to 850 °C with a maximum temperature at 479 °C (Figure 4c), indicating that the formation of HCHO was significantly promoted under hydrogen. This was because hydrogen enhanced the cleavage of lignin side chains and promoted the reduction of acids (such as formic acid) to form more HCHO.

#### 3.3.8. Methanol (CH_3_OH)

The detected evolution temperature ranges of CH_3_OH were 361–595, 395–537, and 299–778 °C (Figure 4d) for Ar, CO_2_, and H_2_ atmospheres, respectively. The cracking of aromatic methoxy (CH_3_O–Ar) and aliphatic –CH_2_OH groups were responsible for the formation of CH_3_OH [39,40]. The formation of CH_3_OH was suppressed under carbon oxidize but significantly promoted under hydrogen compared to argon atmosphere, which was the evolution behavior of HCHO. This phenomenon can be attributed to the oxidizing effects of carbon oxides and the reducing effects of hydrogen.

#### 3.3.9. Hydrogen Sulfide (H_2_S)

Sulfur is introduced to lignin through delignification reaction during a kraft pulping process, which results in sulfur presenting in lignin macromolecular structure as sulfate ions, elemental sulfur, adsorbed polysulfide, and/or organically bound sulfur. Lignin in general contains 2 to 3% sulfur after its kraft pulping process [46], but, kraft lignin used in this experiment contained less than 0.1% sulfur because the intensive acid purification step was applied in its production process. During a depolymerization process of lignin, sulfur is released in gas phases as dimethylsulfide (CH_3_SCH_3_), sulfur vapor, carbon disulfide (CS_2_), carbonyl sulfide (COS), and/or hydrogen sulfide (H_2_S) [47].

The trend of *m*/*z* 34 ion current was monitored as H_2_S evolution during the decomposition of kraft lignin under three different atmospheres (Figure 5). Under argon atmosphere, H_2_S evolution was observed in the temperature range from 210 to 646 °C with a maximum temperature of 341 °C. The evolution profile of H_2_S under CO_2_ atmosphere was similar to the one under Ar atmosphere, while the evolution peak shifted to a lower temperature of 316 °C. Two H_2_S evolution peaks were observed during the thermal decomposition of kraft lignin under a hydrogen flow, that is, the first peak was at 305 °C, which was attributed by the depolymerization process, and the second one was at 949 °C; this peak was assigned to the reduction reaction of sulfur vapor (S + H_2_ → H_2_S) released from the depolymerization process. The presence of hydrogen significantly promoted the depolymerization of lignin and the reduction reaction of sulfur element in lignin.

### 3.4. Liquid Phase Analysis

The composition of liquid products collected from the thermal decomposition of kraft lignin under three different atmospheres are summarized in Table 2. The products were mainly aqueous phases, and the oil phase was negligible. Water was the major product in the final aqueous products, and its weight percentage in the final aqueous products was 90.1%, 85.3%, and 95.5% for Ar, H_2_, and CO_2_ atmospheres, respectively. The organic chemicals in the liquid samples are acetic acid, phenols, ketones alcohols, aldehydes, esters, and some nonidentified compounds.

### 3.5. Analysis and Characterization of Solid Products

***Elemental analysis*:**Table 3 is the summary of the C–H–O–N–S elemental analysis performed on raw and thermally decomposed kraft lignin. The weight percentages of C, H, N, and S in unthermally treated kraft lignin are 65.2 ± 0.2%, 6.1 ± 0.2%, 0.1 ± 0.05%, and 0.8 ± 0.2%, respectively. The solid product of kraft lignin thermally treated under Ar has the lowest carbon content, while the one under CO_2_ has the highest. Hydrogen weight percentages in solid products of kraft lignin thermally treated under Ar, H_2_, and CO_2_ are 0.9 ± 0.1%, 1.0 ± 0.2%, and 0.5 ± 0.1%, respectively. No nitrogen was found in solid products of kraft lignin thermally treated. Sulfur was detected only from the solid product of kraft lignin thermally decomposed under argon flow. Oxygen contents are 1.8 ± 0.5% (under argon), 1.2 ± 0.3% (under hydrogen), and 1.5 ± 0.3% (under CO_2_) for solid products of kraft lignin thermally treated under Ar, H_2_, and CO_2_, respectively.

***BET surface area*:** Surface area analysis results indicate that the solid product of kraft lignin thermally treated under argon had the lowest surface area of 11.3 m^2^/g, while the active atmosphere of CO_2_ yielded the highest BET surface area of 183.9 m^2^/g, followed by H_2_, yielding the surface area of 98.5 m^2^/g.

**Figure 6:**
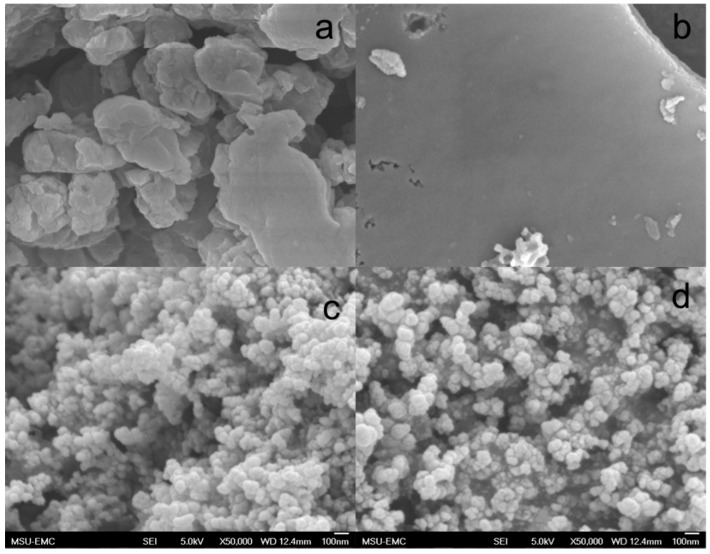
SEM images of raw kraft lignin (**a**) and solid products of kraft lignin thermally decomposed at 1000°C under three different atmospheres of Ar (**b**), CO_2_ (**c**), and H_2_ (**d**).

***Morphology*:**Figure 6 shows SEM images of raw kraft lignin (Figure 6a) and solid products (Figure 6b–d) of kraft lignin thermally decomposed under three different atmospheres. The raw kraft lignin consisted of 1–2-µm particles. The surface of kraft lignin thermally decomposed under argon flow (Figure 6b) was smoother and cleaner. The solid product of kraft lignin thermally decomposed under CO_2_ atmosphere (Figure 6c) was composed of sphere-shaped particles with their sizes ranging from 50 to 80 nm. Figure 6d shows the solid product of kraft lignin thermally decomposed under hydrogen atmosphere also had a large number of sphere-shaped nanoparticles with their sizes ranging from 50 to 100 nm.

## 4. Conclusions

The effects of three different atmospheres of Ar, CO_2_, and H_2_ on the products yielded from the process of the thermal decomposition of kraft lignin were examined by a temperature-programmed desorption-mass spectrometry (TPD-MS) system. Experimental results indicated that the thermal decomposition of kraft lignin under Ar atmosphere yielded the highest solid carbon products, while reactive CO_2_ and H_2_ atmospheres yielded the highest gas and liquid products, respectively. CO_2_ mainly affected the thermal decomposition of kraft lignin at the high temperature range from 650 to 1000 °C, where solid carbon was gasified to CO. Hydrogen was consumed at the temperature range from 205 to 810 °C to form more CH_4_, C_6_H_6_, HCHO, C_6_H_5_OH, H_2_S, CH_3_OH, and tars. Acetic acid, phenols, ketones, alcohols, aldehydes, and esters were detected in liquid products and more organic chemicals were produced under hydrogen atmosphere compared to the other two atmospheres. Solid carbon materials produced under reactive atmospheres (CO_2_ or H_2_) had higher surface areas. Solid products yielded under argon had the lowest carbon content and the highest oxygen content, while ones produced under hydrogen and carbon dioxide atmospheres contained more carbon. Solid products of kraft lignin thermally decomposed under CO_2_ or H_2_ atmosphere were composed of sphere-shaped nanoparticles.

## Figures and Tables

**Table 1:** Summary of gas/volatile evolution temperature ranges and peak temperatures of thermal decomposition of kraft lignin under different atmospheres.

Gas/Volatile	Gas Atmosphere		
	Argon	Hydrogen °C	Carbon Dioxide
H_2_	522–1000 (722)	205–527 (403) * 527–800 (568) * 800–1000	821 (522–1000)
CO_2_	185–583 (407) 583–791 (642)	162–554 (371) 654–770 (600)	585–1000 *
CH_4_	236–532 (455) 532–915 (567)	268–495 (455) 495–688 (615) 688–1000 (745)	266–443 (404) 445–920 (495)
CO	246–679 (422) 679–1000 (773)	155–780 (495) 780–1000 (910)	279–547 (535) 547–1000 (1000)
C_6_H_5_OH	338–610 (473)	239–854 (462)	345–600 (445)
C_6_H_6_	528–938 (697)	510–799 (718) 799–1000	656–1000 (828)
HCHO	329–678 (488)	249–850 (479)	390–601 (482)
CH_3_OH	361–595 (464)	299–778 (443)	395–537 (455)
H_2_S	210–646 (341)	199–656 (305) 704–1000 (949)	228–645 (316)

Note: “*” means values in parentheses are gas consumption.

**Table 2:** Summary of the composition of liquid products (wt %) collected from the thermal decomposition of kraft lignin under three different atmospheres.

Aqueous Phase Components	Ar	H_2_	CO_2_
Water content	90.1	85.3	95.5
Compounds in aqueous phase			
Acetic acid	27.8	25.5	29.1
Acetone	8.5	8.6	9.1
Hydroxyacetaldehyde	6.7	6.6	5.7
Methanol	2.5	4.5	2.1
Phenols	24.7	27.6	24.3
Other acids	8.5	6.1	10.7
Other alcohols	2.7	2.9	1.5
Other ketones	5.9	5.8	5
Other aldehydes	4.3	4.8	4
Esters	1.1	0.8	1.3
Nonidentified	7.3	6.8	7.2

**Table 3:** Summary of weight percentages (wt %) of C, H, O, N, and S in untreated kraft lignin and solid products of kraft lignin thermally decomposed under argon, hydrogen, and carbon dioxide at 1000 °C for 1 h.

Thermally Treated Condition	C	H	O	N	S
Untreated	65.2 ± 0.2	6.1 ± 0.2	27.4 ± 0.9	0.1 ± 0.05	0.8 ± 0.2
Under Ar	92.3 ± 0.7	0.9 ± 0.1	1.8 ± 0.5	-	0.1 ± 0.1
Under H_2_	96.3 ± 0.5	1.0 ± 0.2	1.2 ± 0.3	-	-
Under CO_2_	97.5 ± 0.5	0.5 ± 0.1	1.5 ± 0.3	-	-
